# Supplementary material for: Hybrid Models and Biological Model Reduction with PyDSTool
Source: PLoS Comput Biol. 2012 Aug 9;8(8):e1002628. doi: 10.1371/journal.pcbi.1002628 (PMC3415397; doi:10.1371/journal.pcbi.1002628)
Supplement: Text S4 — Complete source code for the PyDSTool package (version 0.88.120504). Includes API documentation and help files linking to web pages. This file is identical to the current public release on Sourceforge.net. (ZIP) [file pcbi.1002628.s004.zip › PyDSTool/html/PyDSTool.scipy_ode-pysrc.html]

xml version="1.0" encoding="ascii"?


PyDSTool.scipy\_ode


| Home | Trees | Indices | Help | PyDSTool |
| --- | --- | --- | --- | --- |

Package PyDSTool ::
Module scipy\_ode

[hide private]

[frames] | no frames]

# Source Code for Module PyDSTool.scipy\_ode

```
  1  # This is a trivially-adapted version of the original SciPy integrate code.
 
  2  # Changes: (1) removed the annoying print statement whenever
 
  3  #       the ODE code is 'found', which is unwanted screen output during
 
  4  #       computations.
 
  5  #   (2) Used new-style classes.
 
  6  #   (3) Changed a lambda occurrence in one of the methods to use noneFn instead.
 
  7  #-- Rob Clewley, March 2005, June 2008.
 
  8  
 
  9  #Original Author: Pearu Peterson
 
 10  #Date:   3 Feb 2002 (Revision: 1.2)
 
 11  """
 
 12  User-friendly interface to various numerical integrators for solving a
 
 13  system of first order ODEs with prescribed initial conditions:
 
 14  
 
 15         d y(t)[i]
 
 16         ---------  = f(t,y(t))[i],
 
 17          d t
 
 18  
 
 19         y(t=0)[i] = y0[i],
 
 20  
 
 21  where i = 0, ..., len(y0) - 1
 
 22  
 
 23  Provides:
 
 24    ode  - a generic interface class to numeric integrators. It has the
 
 25           following methods:
 
 26             integrator = ode(f,jac=None)
 
 27             integrator = integrator.set_integrator(name,**params)
 
 28             integrator = integrator.set_initial_value(y0,t0=0.0)
 
 29             integrator = integrator.set_f_params(*args)
 
 30             integrator = integrator.set_jac_params(*args)
 
 31             y1 = integrator.integrate(t1,step=0,relax=0)
 
 32             flag = integrator.successful()
 
 33  
 
 34  Supported integrators:
 
 35    vode - Variable-coefficient Ordinary Differential Equation solver,
 
 36           with fixed-leading-coefficient implementation.
 
 37           It provides implicit Adams method (for non-stiff problems)
 
 38           and a method based on backward differentiation formulas (BDF)
 
 39           (for stiff problems).
 
 40           Source: http://www.netlib.org/ode/vode.f
 
 41           This integrator accepts the following pars in
 
 42           set_integrator() method of the ode class:
 
 43             atol=float|seq
 
 44             rtol=float|seq
 
 45             lband=None|int
 
 46             rband=None|int
 
 47             method='adams'|'bdf'
 
 48             with_jacobian=0|1
 
 49             nsteps = int
 
 50             (first|min|max)_step = float
 
 51             order = int        # <=12 for adams, <=5 for bdf
 
 52  """ 
 53  """
 
 54  XXX: Integrators must have:
 
 55  ===========================
 
 56  cvode - C version of vode and vodpk with many improvements.
 
 57    Get it from http://www.netlib.org/ode/cvode.tar.gz
 
 58    To wrap cvode to Python, one must write extension module by
 
 59    hand. Its interface is too much 'advanced C' that using f2py
 
 60    would be too complicated (or impossible).
 
 61  
 
 62  How to define a new integrator:
 
 63  ===============================
 
 64  
 
 65  class myodeint(IntegratorBase):
 
 66  
 
 67      runner = <odeint function> or None
 
 68  
 
 69      def __init__(self,...):                           # required
 
 70          <initialize>
 
 71  
 
 72      def reset(self,n,has_jac):                        # optional
 
 73          # n - the size of the problem (number of equations)
 
 74          # has_jac - whether user has supplied its own routine for Jacobian
 
 75          <allocate memory,initialize further>
 
 76  
 
 77      def run(self,f,jac,y0,t0,t1,f_params,jac_params): # required
 
 78          # this method is called to integrate from t=t0 to t=t1
 
 79          # with initial condition y0. f and jac are user-supplied functions
 
 80          # that define the problem. f_params,jac_params are additional arguments
 
 81          # to these functions.
 
 82          <calculate y1>
 
 83          if <calculation was unsuccesful>:
 
 84              self.success = 0
 
 85          return t1,y1
 
 86  
 
 87      # In addition, one can define step() and run_relax() methods (they
 
 88      # take the same arguments as run()) if the integrator can support
 
 89      # these features (see IntegratorBase doc strings).
 
 90  
 
 91  if myodeint.runner:
 
 92      IntegratorBase.integrator_classes.append(myodeint)
 
 93  """ 
 94  
 
 95  __all__ = ['ode'] 
 96  __version__ = "$Id: ode.py,v 1.2 2003/12/13 13:44:49 pearu Exp $" 
 97  
 
 98  import scipy 
 99  from numpy import asarray, array, zeros, sin 
100  import re, types, sys 
101  
 
102  # use this to replace a lambda expression in the vode calling code
 


103 -def noneFn():


104      return None

105  
 
106  
 


107 -class ode(object):


108      """\
 
109  ode  - a generic interface class to numeric integrators. It has the
 
110    following methods:
 
111      integrator = ode(f,jac=None)
 
112      integrator = integrator.set_integrator(name,**params)
 
113      integrator = integrator.set_initial_value(y0,t0=0.0)
 
114      integrator = integrator.set_f_params(*args)
 
115      integrator = integrator.set_jac_params(*args)
 
116      y1 = integrator.integrate(t1,step=0,relax=0)
 
117      flag = integrator.successful()
 
118  
 
119    Typical usage:
 
120      r = ode(f,jac).set_integrator('vode').set_initial_value(y0,t0)
 
121      t1 = <final t>
 
122      dt = <step>
 
123      while r.successful() and r.t < t1:
 
124          r.integrate(r.t+dt)
 
125          print r.t, r.y
 
126    where f and jac have the following signatures:
 
127      def f(t,y[,arg1,..]):
 
128          return <f(t,y)>
 
129      def jac(t,y[,arg1,..]):
 
130          return <df/dy(t,y)>
 
131      """ 
132  
 


133 -    def __init__(self,f,jac=None):


134          """Define equation y' = f(y,t) where (optional) jac = df/dy.
 
135          User-supplied functions must have the following signatures:
 
136          def f(t,y,...):
 
137              return <f(t,y)>
 
138          def jac(t,y,...):
 
139              return <jac(t,y)>
 
140          where ... means extra pars that can be set with
 
141            set_(f|jac)_params(*args)
 
142          methods.
 
143          """ 
144          self.stiff = 0 
145          self.f = f 
146          self.jac  = jac 
147          self.f_params = () 
148          self.jac_params = () 
149          self.y = [] 
150          self._integrator = None    # R.H.C. added

151  
 


152 -    def set_initial_value(self,y,t=0.0):


153          """Set initial conditions y(t) = y.""" 
154          if type(y) in [types.IntType,types.FloatType]: 
155              y = [y] 
156          n_prev = len(self.y) 
157          self.y = asarray(y,'d') 
158          self.t = t 
159          if not n_prev: 
160              self.set_integrator('') # find first available integrator 
161          self._integrator.reset(len(self.y), self.jac is not None) 
162          return self

163  
 


164 -    def set_integrator(self,name,**integrator_params):


165          """Set integrator by name.""" 
166          integrator = find_integrator(name) 
167          if integrator is None: 
168              print 'No integrator name match with %s or is not available.'\
 
169                    %(`name`) 
170          else: 
171              self._integrator = integrator(**integrator_params) 
172              if not len(self.y): 
173                  self.t = 0.0 
174                  self.y = array([0.0],'d') 
175              self._integrator.reset(len(self.y),self.jac is not None) 
176          return self

177  
 


178 -    def integrate(self,t,step=0,relax=0):


179          """Find y=y(t), set y as an initial condition, and return y.""" 
180          if step and self._integrator.supports_step: 
181              mth = self._integrator.step 
182          elif relax and self._integrator.supports_run_relax: 
183              mth = self._integrator.run_relax 
184          else: 
185              mth = self._integrator.run 
186          self.y,self.t = mth(self.f,self.jac or noneFn,
 
187                              self.y,self.t,t,
 
188                              self.f_params,self.jac_params) 
189          return self.y

190  
 


191 -    def successful(self):


192          """Check if integration was successful.""" 
193          try: self._integrator 
194          except AttributeError: self.set_integrator('') 
195          return self._integrator.success==1

196  
 


197 -    def set_f_params(self,*args):


198          """Set extra-pars for user-supplied function f.""" 
199          self.f_params = args 
200          return self

201  
 


202 -    def set_jac_params(self,*args):


203          """Set extra-pars for user-supplied function jac.""" 
204          self.jac_params = args 
205          return self

206  
 
207  #############################################################
 
208  #### Nothing interesting for an end-user in what follows ####
 
209  #############################################################
 
210  
 


211 -def find_integrator(name):


212      for cl in IntegratorBase.integrator_classes: 
213          if re.match(name,cl.__name__,re.I): 
214  ##            print 'Found integrator',cl.__name__
 
215              return cl

216  
 


217 -class IntegratorBase(object):


218  
 
219      runner = None            # runner is None => integrator is not available 
220      success = None           # success==1 if integrator was called successfully 
221      supports_run_relax = None 
222      supports_step = None 
223      integrator_classes = [] 
224  
 


225 -    def reset(self,n,has_jac):


226          """Prepare integrator for call: allocate memory, set flags, etc.
 
227          n - number of equations.
 
228          has_jac - if user has supplied function for evaluating Jacobian.
 
229          """

230  
 


231 -    def run(self,f,jac,y0,t0,t1,f_params,jac_params):


232          """Integrate from t=t0 to t=t1 using y0 as an initial condition.
 
233          Return 2-tuple (y1,t1) where y1 is the result and t=t1
 
234          defines the stoppage coordinate of the result.
 
235          """ 
236          raise NotImplementedError,\
 
237          'all integrators must define run(f,jac,t0,t1,y0,f_params,jac_params)'

238  
 


239 -    def step(self,f,jac,y0,t0,t1,f_params,jac_params):


240          """Make one integration step and return (y1,t1).""" 
241          raise NotImplementedError,'%s does not support step() method' %\
 
242                (self.__class__.__name__)

243  
 


244 -    def run_relax(self,f,jac,y0,t0,t1,f_params,jac_params):


245          """Integrate from t=t0 to t>=t1 and return (y1,t).""" 
246          raise NotImplementedError,'%s does not support run_relax() method' %\
 
247                (self.__class__.__name__)

248  
 
249      #XXX: __str__ method for getting visual state of the integrator
 
250  
 


251 -class vode(IntegratorBase):


252      try: 
253          import scipy.integrate.vode as _vode 
254      except ImportError: 
255          print sys.exc_value 
256          _vode = None 
257      runner = getattr(_vode,'dvode',None) 
258  
 
259      messages = {-1:'Excess work done on this call. (Perhaps wrong MF.)',
 
260                  -2:'Excess accuracy requested. (Tolerances too small.)',
 
261                  -3:'Illegal input detected. (See printed message.)',
 
262                  -4:'Repeated error test failures. (Check all input.)',
 
263                  -5:'Repeated convergence failures. (Perhaps bad'
 
264                  ' Jacobian supplied or wrong choice of MF or tolerances.)',
 
265                  -6:'Error weight became zero during problem. (Solution'
 
266                  ' component i vanished, and ATOL or ATOL(i) = 0.)'
 
267                  } 
268      supports_run_relax = 1 
269      supports_step = 1 
270  
 


271 -    def __init__(self,
 
272                   method = 'adams',
 
273                   with_jacobian = 0,
 
274                   rtol=1e-6,atol=1e-12,
 
275                   lband=None,uband=None,
 
276                   order = 12,
 
277                   nsteps = 500,
 
278                   max_step = 0.0, # corresponds to infinite
 
279                   min_step = 0.0,
 
280                   first_step = 0.0, # determined by solver
 
281                   ):


282  
 
283          if re.match(method,r'adams',re.I): self.meth = 1 
284          elif re.match(method,r'bdf',re.I): self.meth = 2 
285          else: raise ValueError,'Unknown integration method %s'%(method) 
286          self.with_jacobian = with_jacobian 
287          self.rtol = rtol 
288          self.atol = atol 
289          self.mu = lband 
290          self.ml = uband 
291  
 
292          self.order = order 
293          self.nsteps = nsteps 
294          self.max_step = max_step 
295          self.min_step = min_step 
296          self.first_step = first_step 
297          self.success = 1

298  
 


299 -    def reset(self,n,has_jac):


300          # Calculate pars for Fortran subroutine dvode.
 
301          if has_jac: 
302              if self.mu is None and self.ml is None: 
303                  miter = 1 
304              else: 
305                  if self.mu is None: self.mu = 0 
306                  if self.ml is None: self.ml = 0 
307                  miter = 4 
308          else: 
309              if self.mu is None and self.ml is None: 
310                  if self.with_jacobian: 
311                      miter = 2 
312                  else: 
313                      miter = 0 
314              else: 
315                  if self.mu is None: self.mu = 0 
316                  if self.ml is None: self.ml = 0 
317                  if self.ml==self.mu==0: 
318                      miter = 3 
319                  else: 
320                      miter = 5 
321          mf = 10*self.meth + miter 
322          if mf==10: 
323              lrw = 20 + 16*n 
324          elif mf in [11,12]: 
325              lrw = 22 + 16*n + 2*n*n 
326          elif mf == 13: 
327              lrw = 22 + 17*n 
328          elif mf in [14,15]: 
329              lrw = 22 + 18*n + (3*self.ml+2*self.mu)*n 
330          elif mf == 20: 
331              lrw =  20 +  9*n 
332          elif mf in [21,22]: 
333              lrw = 22 + 9*n + 2*n*n 
334          elif mf == 23: 
335              lrw = 22 + 10*n 
336          elif mf in [24,25]: 
337              lrw = 22 + 11*n + (3*self.ml+2*self.mu)*n 
338          else: 
339              raise ValueError,'Unexpected mf=%s'%(mf) 
340          if miter in [0,3]: 
341              liw = 30 
342          else: 
343              liw = 30 + n 
344          rwork = zeros((lrw,),'d') 
345          rwork[4] = self.first_step 
346          rwork[5] = self.max_step 
347          rwork[6] = self.min_step 
348          self.rwork = rwork 
349          iwork = zeros((liw,),'i') 
350          iwork[4] = self.order 
351          iwork[5] = self.nsteps 
352          iwork[6] = 2           # mxhnil 
353          self.iwork = iwork 
354          self.call_args = [self.rtol,self.atol,1,1,self.rwork,self.iwork,mf] 
355          self.success = 1

356  
 


357 -    def run(self,*args):


358          y1,t,istate = self.runner(*(args[:5]+tuple(self.call_args)+args[5:])) 
359          if istate <0: 
360              print 'vode:',self.messages.get(istate,'Unexpected istate=%s'%istate) 
361              self.success = 0 
362          else: 
363              self.call_args[3] = 2 # upgrade istate from 1 to 2 
364          return y1,t

365  
 


366 -    def step(self,*args):


367          itask = self.call_args[2] 
368          self.call_args[2] = 2 
369          r = self.run(*args) 
370          self.call_args[2] = itask 
371          return r

372  
 


373 -    def run_relax(self,*args):


374          itask = self.call_args[2] 
375          self.call_args[2] = 3 
376          r = self.run(*args) 
377          self.call_args[2] = itask 
378          return r

379  
 
380  if vode.runner: 
381      IntegratorBase.integrator_classes.append(vode) 
382  
 
383  
 


384 -def test1(f):


385  
 
386      ode_runner = ode(f) 
387      ode_runner.set_integrator('vode') 
388      ode_runner.set_initial_value([0.1,0.11,.1]*10) 
389  
 
390      while ode_runner.successful() and ode_runner.t < 50: 
391          y1 = ode_runner.integrate(ode_runner.t+2) 
392          print ode_runner.t,y1[:3]

393  
 


394 -def test2(f, jac):


395      # Stiff problem. Requires analytic Jacobian.
 
396      r = ode(f,jac).set_integrator('vode',
 
397                                    rtol=1e-4,
 
398                                    atol=[1e-8,1e-14,1e-6],
 
399                                    method='bdf',
 
400                                    ) 
401      r.set_initial_value([1,0,0]) 
402      print 'At t=%s  y=%s'%(r.t,r.y) 
403      tout = 0.4 
404      for i in range(12): 
405          r.integrate(tout) 
406          print 'At t=%s  y=%s'%(r.t,r.y) 
407          tout *= 10

408  
 
409  
 


410 -def f1(t,y):


411      a = sin(6*t) 
412      return y*y-a+y

413  
 


414 -def f2(t,y):


415      ydot0 = -0.04*y[0] + 1e4*y[1]*y[2] 
416      ydot2 = 3e7*y[1]*y[1] 
417      ydot1 = -ydot0-ydot2 
418      return [ydot0,ydot1,ydot2]

419  
 


420 -def jac(t,y):


421      jc = [[-0.04,1e4*y[2]          ,1e4*y[1]],
 
422            [0.04 ,-1e4*y[2]-6e7*y[1],-1e4*y[1]],
 
423            [0.0    ,6e7*y[1]           ,0.0]] 
424      return jc

425  
 
426  
 
427  if __name__ == "__main__": 
428      print 'Integrators available:',\
 
429            ', '.join(map(lambda c:c.__name__,
 
430                          IntegratorBase.integrator_classes)) 
431  
 
432      test1(f1) 
433      test2(f2, jac) 
434
```

  


| Home | Trees | Indices | Help | PyDSTool |
| --- | --- | --- | --- | --- |

|  |  |
| --- | --- |
| Generated by Epydoc 3.0.1 on Fri May 4 15:24:25 2012 | http://epydoc.sourceforge.net |
